# The Effectiveness of a Mobile Learning Environment in Improving Psychological Security in Blind Students

**DOI:** 10.1155/2024/7629607

**Published:** 2024-07-08

**Authors:** Amr El Koshiry, Entesar Eliwa, Tarek Abd El-Hafeez, Mohamed Abd Allah Tony

**Affiliations:** ^1^ Department of Curricula and Teaching Methods College of Education King Faisal University, P.O. Box: 400, Al-Ahsa 31982, Saudi Arabia; ^2^ Faculty of Specific Education Minia University, Minia 61519, Egypt; ^3^ Department of Mathematics and Statistics College of Science King Faisal University, P.O. Box: 400, Al-Ahsa 31982, Saudi Arabia; ^4^ Department of Computer Science Faculty of Science Minia University, Minia 61519, Egypt; ^5^ Computer Science Unit Deraya University Minia University, Minia 61519, Egypt; ^6^ Department of Curriculum Teaching Methods and Education Technology Faculty of Education Arish University, Arish 4551, Egypt

## Abstract

This study aimed to investigate the impact of the Edmodo mobile learning environment on promoting psychological security among university students with visual impairments, at both the undergraduate and postgraduate levels. The researchers employed a combination of descriptive and quasiexperimental methodologies. The primary study sample consisted of 20 visually impaired students from Beni Suef University, divided equally between an experimental group (10 students) and a control group (10 students). To achieve the research objectives, the Psychological Security Scale was utilized and the experimental group received an intervention involving the implementation of a mobile learning environment using Edmodo. The data analysis revealed a statistically significant difference between the experimental and control groups in the postassessment, with the experimental group demonstrating an elevated sense of psychological security. Furthermore, the experimental group showed significant improvements in the pre- and postassessments, favoring the latter, with a standard score of 3.781. No significant differences were observed between the postassessment and the follow-up evaluation of the experimental group, with a standard score of 0.471, indicating the continuous effectiveness of the Edmodo mobile learning environment in enhancing the psychological security of visually impaired university students. This efficacy was sustained even one month after the student's graduation, as evidenced by the follow-up assessment.

## 1. Introduction

Individuals with visual impairments are vital members of our society, proving time and again that they can accomplish incredible feats despite their physical challenges. The 2018 Rights of Persons with Disabilities Act validates their rights to accessible higher education, including the ability to partake in distance learning [1]. Technology, especially mobile learning environments enabled via handheld devices, plays a key role, in aiding these visually impaired students to meet several educational objectives. These devices provide a convenient way for them to process and distribute knowledge [2].

Visually impaired students have proven adept at using digital media, including smartphones and computers, with the help of screen readers. This transition from traditional to digital spaces has significantly improved their communication and learning, spotlighting the importance of digital literacy for this group. The internet offers them a plethora of services, as guaranteed by the law, including education, employment, banking services, and social media connectivity, necessitating heightened awareness and security against potential cyber threats [3].

However, psychological insecurity is a significant challenge faced by the visually impaired demographic, caused by societal attitudes and their disability. Studies suggest that using technology, such as mobile phones and applications, can positively contribute to the psychosocial security of visually impaired individuals. Thus, mobile learning environments offer a valuable opportunity. They provide education and training resources that surpass traditional time, space, or physical constraints. This approach can effectively enhance the psychological security and life satisfaction of visually impaired students [4, 5].

The need to take advantage of mobile learning environments is evident to suit a blind student. Opportunities for education and training in the blind's hands create the barriers of time, space, and disability, please him in education, and work to improve his psychological security, a fundamental and influential component of the lives of the blind.

### 1.1. Problem Statement

The current research problem stemmed from several sources, including

1. Field observation: The researcher noted that many blind students had smartphones they used for a variety of purposes, including communication social networking, digital commerce, entertainment, education, scientific research, and training, many of whom also relied on mobile phones as an alternative to visible facilities in some of their affairs and life activities Until their daily dependence on those phones became essential, it cannot be overlooked, what some studies have pointed out: [3, 6–8]. Poor psychosocial security was observed in some of the university's blind students, which was reflected in the complaints and suffering of these students and their exposure to attempts to penetrate and fraud in the absence of their sense of sight.
2. Results of previous studies: These modern technologies are no longer merely recreational methods used by individuals. In fact, there have been recent tendencies taken by many educational institutions at different stages to use these modern technologies to develop their children's skills and abilities. Hence, mobile learning environments have relied on modern techniques that can be used in education and training processes for students in general and blind in particular because of their ability to acquire different skills in a faster way, as noted by some recent studies [9, 10], which noted the effectiveness of the mobile learning environment in providing students with visual disabilities with some skills and values. Some studies also indicated that the blind did not feel psychosocial from mobile learning environments [11, 12], and some of these studies have also suggested the possibility of developing their sense of psychological security by following appropriate interventions and preparing plans and programs to achieve this.
3. Exploratory study: Through the abovementioned field observations and the results of previous studies, an exploratory study was done by preparing a simple note card with psychosocial security presented to some students of the research community, and the responses of blind students indicated their poor psychosocial security. The results of his studies [7, 9] refer to the effectiveness of mobile learning environments in developing many of the skills of blind students. On the other hand, many references indicated that there is a lack of psychosocial security among blind people and recommended that their level of psychosocial security be improved.

Thus, the researcher found that blind students are in dire need of improving their psychosocial security through the use of electronic technologies and the development of this use on a scientific basis so that it becomes a training and educational entity through the design of a learning environment based on mobile learning using the Edmodo platform through which to address the difficulties and problems facing traditional learning environments of blind students and investing such modern technological environments in providing attractive educational activities that contribute to improving the psychosocial security of blind students.

### 1.2. Research Questions

Based on the foregoing, the problem of research has been initiated to answer the following main question:

What is the effectiveness of the mobile learning environment in improving the psychosocial security of students blind to university education (undergraduate and postgraduate)?

The following subquestions emerged from the chairman's question:

1. What are the differences between the average grades of the members of the experimental and control groups in the dimensional measurement on the psychosecurity scale (dimensions and overall grade)?
2. What are the differences between the average grades of the experimental group in tribal and postmetric measurements on the psychosecurity scale (dimensions and overall grade)?
3. What are the differences between the average grades of the experimental group in dimensional and tracking measurements on the psychosecurity scale (dimensions and overall grade)?

### 1.3. Research Objectives

The research aims at improving the psychosocial security of students blind to university education (undergraduate and postgraduate) through

1. Detection of differences between the average grades of the members of the experimental and control groups in the dimensional measurement on the psychosecurity scale (dimensions and overall grade).
2. Detection of differences between the averages of the grades of the experimental group in tribal and postgraduate measurements on the scale of psychosecurity (dimensions and overall grade).
3. Detection of differences between the average grades of the experimental group in dimensional and tracking measurements on the psychosecurity scale (dimensions and overall grade).

This research holds both theoretical and practical significance. Theoretically, it addresses the academic needs of visually impaired students, a notably under-researched group in higher education, and aims to ensure their right to psychological security, a common area of concern for individuals with visual impairments. It also fills a gap in studies on the impact of mobile learning environments on the psychosocial well-being of these students. Practically, the study presents an educational model using the Edmodo platform to create an accessible mobile learning environment for visually impaired students, enhancing their skills and supporting their rights as outlined in the Rights of Persons with Disabilities Act No. 10 of 2018.

This research focuses on the psychosocial impact of mobile learning via Edmodo for blind university students, involving 20 participants from Beni Suef University. It was conducted in the second semester of the 2021-2022 academic year across various colleges. Operational definitions within the study include the mobile learning environment as an online educational space using Edmodo, compatible with speech programs for accessibility. Psychosocial security is defined as feelings of safety and comfort measured by a student's score on a psychosocial security scale. Blind students are defined as those with significant vision impairment enrolled in undergraduate or graduate degrees at Beni Suef University, without additional disabilities.

## 2. Related Work

### 2.1. Theoretical Framework and Previous Studies

The theoretical framework included four axes, which are dealt with in some detail as follows.

#### 2.1.1. First Axis: Visual Disability

According to the World Health Organization, a person is considered blind if their vision is less than 3/60 meters, meaning they can only perceive what a person with normal vision can see at 60 meters when they are only 3 meters away. Globally, about 2.2 billion people suffer from visual impairments [13], and in Egypt, 4.73% of the population has visual disabilities [14].

Blind individuals may face various challenges, including limited mobility and the need to exert more energy to perceive their environment, which may lead to feelings of insecurity and difficulty in social adaptation. Despite these challenges, many visually impaired individuals pursue higher education, supported by laws ensuring their right to education [15].

The use of supportive educational and technological programs has been crucial in helping visually impaired people adapt to the digital world and maintain psychological security [11]. Therefore, continuous development of these resources is essential to ensure they can lead fulfilling lives.

Visual impairment encompasses a spectrum of conditions affecting sight, ranging from minor issues like squinting to complete blindness [16]. Globally, visual impairment poses a significant challenge, with approximately 2.2 billion individuals affected to varying degrees, including 1 billion who are blind [17]. Among the elderly population in China, the prevalence of visual impairment is 8.10%, with 1.97% experiencing both visual and hearing impairments concurrently [18]. However, accurately assessing visual impairment presents difficulties, particularly in cases where visual acuity remains intact but visual fields are limited. This underscores the necessity for comprehensive guidelines that address both aspects when certifying disabilities. In conditions such as Neuromyelitis Optica Spectrum Disorder (NMOSD), visual impairment can arise from acute attacks of optic neuritis, underscoring the importance of prognostic models for predicting and managing the risks associated with visual impairment [19].

#### 2.1.2. Second Axis: Mobile Learning Environments

Mobile learning, a form of e-learning, is facilitated by wireless devices like mobile phones and tablets [20]. It is a flexible and interactive mode of education that transcends time and space [21]. Key characteristics include mobility, adaptability, and availability [22]. Mobile learning promotes student-centered processes, enhances interactions, and provides lifelong learning opportunities [23]. Various strategies support mobile learning, including Attitudinal Learning Environments Strategies, Mobile Participatory Learning Methods, and Virtual Community of Practice [24]. Importantly, mobile learning has been instrumental for the visually impaired, offering nontraditional learning environments and helping them perform daily tasks with ease [25]. Blind use for mobile phone applications: [7] an analysis of a series of lessons on the use of blind people for mobile applications and classified them into seven themes.

Accessibility in mobile apps is crucial for blind users to ensure equal usability. Blind individuals utilize mobile apps to support their independence in daily activities and learning, with tools like Braille-based applications. Auxiliary devices like screen readers and wearables help them overcome challenges and enhance their quality of life. However, they face difficulties with limited gesture mobility and require familiar gestures for technologies like speech interaction on touchscreens. Screen layout also presents challenges, as blind users may struggle with identifying and interacting with elements on touch interfaces, leading to accidental touches and incorrect patterns. Voice guidance is essential for their daily interaction with apps, providing auditory cues. Lastly, navigation is a significant issue, especially with complex graphical interfaces, and solutions like sound-based guidance, digital maps, image sonication, and multimodal outputs like sound and vibration are recommended to improve their mobility. In the research provided, mobile learning environments are explored for their ability to provide varied learning opportunities through the use of mobile technologies [26]. These environments involve the creation of scenarios, the generation of learning materials, and their distribution via devices such as smartphones and tablets [27]. They empower learners to partake in creative tasks, social interactions, and content management regardless of time or location [28]. When designing mobile learning applications for educational purposes, future developers must address specific challenges [29]. In essence, mobile learning environments harness technology to support learning across formal, informal, and nonformal settings, highlighting the importance of seamless access to knowledge and interactive learning experiences.

#### 2.1.3. Third Axis: Edmodo Educational Platform

Edmodo, launched in 2008 by Nick Borg and Jeff O'Hara, is a prominent social learning platform often referred to as the “Facebook for Education.” It provides a secure, user-friendly environment for teachers, students, and parents to interact and engage in the educational process [30]. The platform is free and facilitates global communication, classroom management, and content sharing, similar to social networks like Facebook and Twitter [31]. It offers a digital learning space that enhances participation and skill acquisition and transcends geographical and temporal barriers. Edmodo combines features of e-content management systems with social networking, offering tools such as a grading system, an archive system, and app integration. Its interface is intuitive, resembling Facebook, which simplifies use for students. Teachers can create virtual classrooms, conduct discussions, exchange files, and communicate with parents about student performance. Edmodo has transformed education by supporting online collaboration and has been particularly effective in medical instruction [32, 33]. Research suggests that it improves student engagement, promotes responsible learning, and positively impacts academic performance and attitudes, particularly in science. It also aids in assessing reading comprehension, reducing cheating, and encouraging independent student work [34].

#### 2.1.4. Fourth Axis: Psychological Security

Psychosocial security is a vital concept in positive psychology, integral to an individual's personality and mental health [35]. It reflects the quality of relationships within the family, which can either contribute to or detract from a child or adolescent's sense of psychological security [36, 37]. Recognized by Maslow as a fundamental human need, psychosocial security is associated with feelings of happiness, satisfaction, and safety and is essential from birth through all life stages. For blind individuals, psychosocial security is particularly crucial, as societal attitudes and limited early experiences can adversely affect their psychological well-being, potentially leading to antisocial behavior and psychological issues. Psychosocial security influences educational achievement, intellectual development, and social attitudes like tolerance and acceptance. Conversely, insecurity can manifest as negative emotions and psychosomatic symptoms [38, 39]. A supportive family environment can significantly bolster the psychosocial security of blind individuals. In educational settings, ensuring psychological security for students with disabilities is key to their success [40, 41]. Factors such as personal safety, social inclusion, emotional well-being, and self-esteem are critical to their psychological security [42]. Training in social skills and effective communication can enhance psychosocial security for blind students, highlighting the importance of addressing these needs for their overall welfare and academic achievement [43].  Table 1 represents the recent Studies on Mobile Learning Environments for Individuals with Visual Disabilities.

#### 2.1.5. Educational Theories Guiding Current Research

The current research is underpinned by educational theories that highlight the significance of engaging in activities for fostering learner positivity, active participation, and the pursuit of knowledge. These theories support the use of a mobile learning environment to build constructive and meaningful experiences [48, 49].

Key theories guiding the research include

1. Constructive theory: This posits that learners actively construct their knowledge and understanding, integrating new information with what they already know. It emphasizes understanding, application, thinking, and analysis over mere memorization and repetition [50].
2. Cognitive theory: This centers on the learner's active participation and considers individual learning styles and cognitive processes. It focuses on how knowledge is stored and retrieved in the learner's memory [51].
3. Active learning theory: This suggests that e-activity is a system with interrelated components that evolve, especially with advancements in the Internet and communications. It encourages learner participation and innovation in activities [52].

The researcher utilizes these theories to create diverse electronic activities within the mobile learning environment. These activities are designed to cater to the varied learning styles of blind students, stimulate mental processes, aid in knowledge retention, and ensure students can recall information effectively in different educational contexts. Guidance is provided to enable students to perform these activities efficiently.

#### 2.1.6. Research Assumptions

Drawing from theoretical foundations and previous research, the study proposed the following hypotheses:

1. The experimental group's average scores on the psychosocial security scale (considering all aspects and the total score) during the initial assessment will be significantly higher than those of the control group.
2. The experimental group's average scores on the psychosocial security scale will show significant improvement when comparing the initial assessment to the subsequent assessment, with the latter scores expected to be higher.
3. There will be no significant difference in the average scores of the experimental group on the psychosocial security scale when comparing the subsequent assessment to the follow-up assessment, indicating stability across all aspects and the total score.

## 3. Materials and Methods

### 3.1. Research Curriculum and Sample Size

The research employed a descriptive approach to characterize and assess the psychosocial security among visually impaired university students, both at undergraduate and postgraduate levels. Additionally, a quasiexperimental design was used to examine the effect of the independent variable, which is the “Mobile learning environment using the Edmodo educational platform,” on the dependent variable “psychosocial security.” This involved pretesting both the experimental and control groups, ensuring they were comparable, and then implementing the educational design through the mobile learning environment with the Edmodo platform for the experimental group. The outcomes were measured by comparing the scores of the experimental and control groups before and after the intervention and by evaluating the experimental group's scores one month after the intervention to assess the lasting impact of the mobile learning environment.

The original community of research (49) is a blind student (male and female) at Beni Suef University in the following colleges: Arts, Science with Special Needs, Media, Alson, and Law, of whom (39) are students and undergraduate students (bachelor's degree and license), and (10) are postgraduate students who are blind (public diploma-private diploma master's degree-doctorate).  Table 2 shows the demographic characteristics of the research community.

A sample of 25 students from the current research community was selected to verify the psychometric characteristics of the research tools; (21) of them are students blind to various undergraduate teams (bachelor's degree), as well as (4) graduate students blind to Beni Suef University.

Basic sample: The basic research sample consisted of 20 students and blind students; of them, 15 were students of various bachelor's and bachelor's degree groups, 5 were students blind to graduate studies at Beni Suef University and divided into two groups; a control group of (10) students, an experimental group of (10) students, and Table 1 show the distribution of the research sample.

### 3.2. The Research Sample Characteristics

1. Blind students enrolled in various undergraduate teams (bachelor's degree and bachelor's degree), as well as blind students enrolled in postgraduate studies (public diploma, private diploma, master's degree, doctorate) at Beni Suef University colleges for the university year 2021-2022. To ensure participants met the World Health Organization's criteria for blindness, which is vision less than 3/60, we checked their medical records and obtained confirmations from certified ophthalmologists to verify their eligibility.
2. Blind students have no total sight and no other disabilities.
3. Blind students with smart mobile phones with technological expertise in handling mobile phones and applications.

Those specifications were available in the current sample of research after the exclusion of those who did not meet those specifications, who were (4) students; (2) of them have poor eyesight, (2) have no technological expertise, and do not have mobile smartphones; thus, the basic sample of the current research consisted of (20) blind students.

#### 3.2.1. The Average Age of the Search Sample

The quasiexperimental design (two sets) was selected where the research contains two groups (control and experimental) with tribal and postgraduate measurement. Each group was composed of 10 students with blind university education as shown in Table 3.  Table 4 shows the parity between the groups (experimental and control) of time and age.

As shown in Table 4,

1. X: They were exposed to the mobile learning environment through the Edmodo platform
2. Y: They were exposed to the measure (tribal and remote) of psychosocial security

#### 3.2.2. Parity between the Two Groups (Experimental and Control)

Table 5 presents a comparative analysis of the experimental and control groups, focusing on the parity between the two in terms of age and time. The table includes detailed statistics such as the number of participants (*N*), arithmetic average, standard deviation, average grades, and total grades for each group. Additionally, it provides the results of statistical tests with values for “*u*” and “*z*,” as well as the indicative level, which helps in understanding the significance of the differences observed between the experimental group and the control group. This comprehensive overview allows for a clear comparison of the two groups' characteristics and outcomes within the context of the study.

Table 5 reveals that there are no significant statistical differences in the average grades of the experimental and control groups over their lifetimes. This demonstrates that the two groups were equivalent at the baseline measurement, setting the stage for a valid systematic intervention. Similarly, as shown in the previous table, the lack of significant differences in the lifetime average grades between the experimental and control groups confirms their equivalence in the baseline measurement, ensuring a proper foundation for the systematic intervention.  Table 6 further illustrates this equivalence between the two groups in terms of psychosocial security.

As shown in Table 6, there are no statistically significant differences between the grading averages of the experimental and control groups in psychosocial security. This indicates their parity in tribal application, thus paving the way for the correct systematic application.

### 3.3. Measurement Tools

First: Psychosecurity Measure (Emotional Reassurance) Preparation [53]:

The objective of this measure is to be used as a codified objective tool in diagnosing psychological security (emotional reassurance) for many diverse clinical groups in both health and disease and the field of persons with disabilities, as well as in psychological and educational research, at all stages of an individual's life cycle from late childhood to old age.

Metric description: Scale items are distributed over four main axes:

1. Psychosocial security associated with an individual's composition and vision for the future (14 items)
2. Psychosocial security associated with an individual's public and practical life (18 items)
3. Psychosocial security associated with an individual's mood (10 items)
4. Psychosocial security associated with one's social relations and social interaction (12 items)

The scale in question consists of 54 items designed to evaluate an individual's psychosocial security. Respondents indicate their level of agreement with each statement using a four-point scale (strongly agree, agree, disagree, strongly disagree). Scoring is differentiated between items 1–19, which are positively oriented towards psychosocial security, and items 20–54, which are negatively oriented. The total possible score ranges from 0 to 162, with various levels of psychosocial security defined within this range.

The scale's reliability was established through various methods, including correlation with a similar scale, internal consistency, and test-retest reliability, yielding high coefficients that confirm its stability and usability. Additionally, the scale's validity was supported by its application to a sample of university students, including a specific validation for use with blind university students. The internal consistency was further examined by correlating each item's score with the total score of its dimension, with results indicating high correlation coefficients, as shown in Table 7.

Pearson coefficients are calculated between the psychosocial dimensions of blind students on the one hand and each dimension of the scale's overall degree on the other; Table 8 shows the psychosecurity scale associations' matrix.

Table 8 indicates that all correlation values are significant at the 0.01 level, which confirms the internal consistency of the measure.

#### 3.3.1. Scale Stability

The stability of the psychosecurity measure in blind students was calculated by applying the measure and reapplying it at an interval of two weeks on the psychometric verification sample, and the correlations between the sample scores were calculated using the Pearson coefficient (Pearson), and all correlations factors of scale dimensions were a function of (0.01) indicating that the scale gives roughly the same results if used more than once under similar circumstances as shown in Table 9.

Table 9 presents a statistically significant correlation between the initial and subsequent applications of the psychosocial security scale's dimensions and its total score. This finding underscores the scale's stability and validates the use of the psychosocial security measure for individuals with visual impairments, confirming its effectiveness in assessing the intended trait.

#### 3.3.2. Alpha-Cronbach Modulus Method

The calculation of the MSM persistence factor involved assessing the Alpha-Cronbach coefficient for each dimension of the scale. All values obtained were deemed acceptable, indicating a satisfactory level of stability. The results are presented in Table 10.

Table 10 shows that stabilization factors are acceptable, giving a good indicator of scale stability, and therefore can be applied.

#### 3.3.3. Third: Proposed Scenario of Quasiexperimental Treatment Material

**Figure 1:**
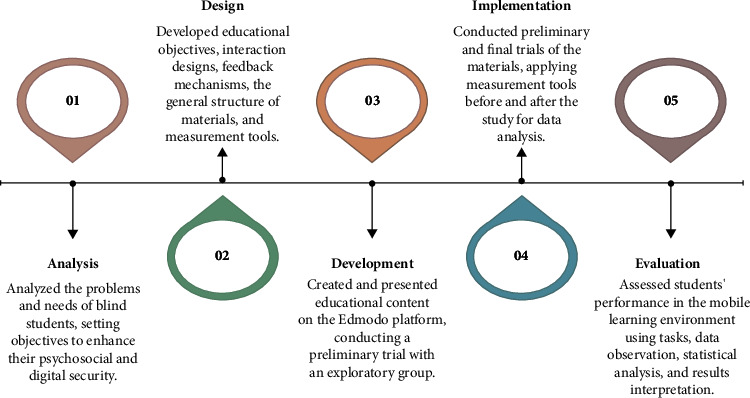
General flowchart steps for the quasiexperimental processing model.

*(1) Edmodo Platform*. We chose the Edmodo platform for the study after assessing various educational platforms, considering their pros and cons, and the specific needs of blind university students. Edmodo was found to provide many necessary features for these students. Blind students in the trial group were assisted in setting up their Edmodo accounts. The ADDIE model, known for its flexibility and relevance, was used for educational design in the research, encompassing five stages: Analysis, Design, Development, Implementation, and Evaluation.  Figure 1 illustrates the sequential steps of the quasiexperimental processing model in the form of a general flowchart.

1. The analysis phase involved identifying the problems and needs of blind students, setting general objectives, analyzing the characteristics and needs of the research group, and identifying obstacles or limitations. Notably, the primary objective was to improve blind students' psychosocial and digital security through designing an appropriate electronic environment for them.
2. In the design phase, the researcher formulated educational objectives, designed interactions and feedback, constructed the general structure of the processing material, and designed the measurement tools.
3. The construction phase concerned the production of the educational content and the processing material necessary for enhancing the research group's psychosocial security. The content was created and presented through the Edmodo platform. This material was subjected to a preliminary trial with an exploratory group.
4. The implementation phase encompassed the preliminary experimentation with the processing material and the final usage of this material. Two measurement tools were used, and their application prestudy and poststudy allowed for efficient data analysis.
5. The evaluation phase assessed the performance of the blind students within the mobile learning environment through tasks, data observation, statistical treatment, and results' interpretation.

Various statistical methods were employed, including calculations of percentages, averages, and standard deviation, the Mann–Whitney test, and the Wilcoxon test. Furthermore, confirmatory factor analysis was executed using the statistical program AMOS 26.

## 4. Results and Discussion

Verification of the results of the first imposition:

The assumption states that “there are statistically significant differences between the average grades of the members of the experimental and control groups in the dimensional measurement of the psychosecurity scale (dimensions and overall grade) in favor of the experimental group.” To test the validity of this assignment, Mann's test was used.  Table 11 shows the results of this assignment.

As indicated in Table 11, there are holistically significant differences at a significance level of 0.01 between the average grades of the experimental and control groups in the psychosocial security scale dimensions. The experimental group has statistically higher grades, validating the fourth proposition. This can be attributed to the lower level of psychosocial security in the control group's students. The lack of sight significantly contributes to their sense of insecurity. Sight is crucial for recognizing potential dangers in one's environment, as indicated in a statement by the Prophet Muhammad, emphasizing sight's protective function. In addition to this, blind students face unique challenges impacting their psychosocial security. The necessity to rely heavily on assistive facilities in academia often leads to feelings of insecurity. Moreover, they need to adapt to digital advancements in educational institutions, which can be particularly challenging in the university context. The requirement for daily interaction with technological media further compounds their sense of insecurity if they struggle to efficiently navigate these platforms.

**Figure 2:**
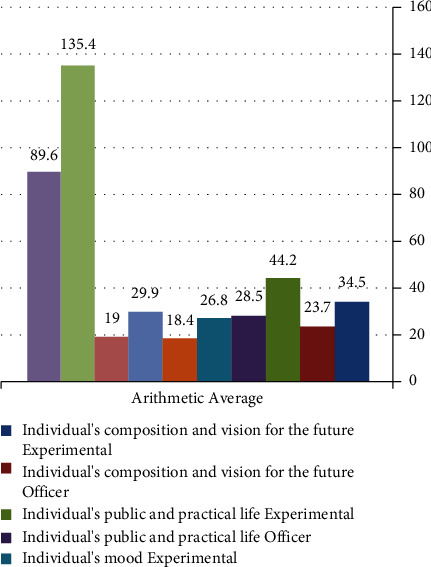
Intermediate scores of experimental and control groups on the psychosecurity scale.

As a result, the control group appears disadvantaged in the psychosocial security scale due to not being exposed to the mobile learning environment, as evident in Figure 2.

Figure 2 illustrates that the students from the experimental group displayed heightened levels of psychosocial security when compared to the control group, noted after the program's application.

The proposition alleged that “significant statistical differences exist between the average grades of the experimental group in pre- and postmeasurements on the psychosocial security scale in favor of the postmeasurements.” The accuracy of this proposition was tested using the Wilcoxon “*W*” test, and the outcomes are showcased in Table 12.

**Figure 3:**
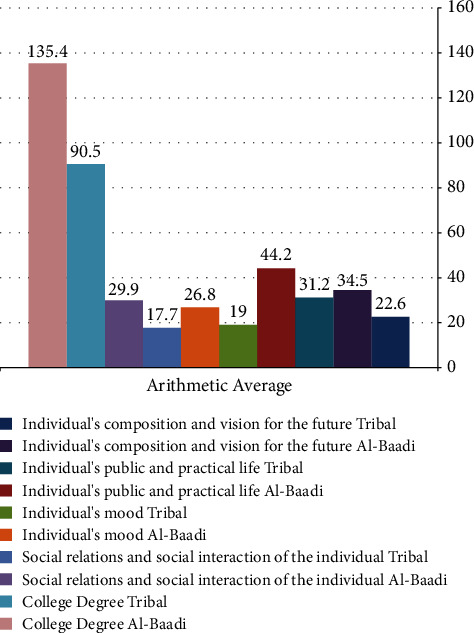
Average scores of tribal and postgraduate measurements in the experimental group on the psychosecurity scale.

Table 12 indicates statistically significant differences at a 0.01 level between the pilot group students' pretest and posttest scores on aspects of the psychosocial security scale. The posttest scores are significantly higher, validating the fifth imposition. This finding aligns with Wally's 2020 study, showing a statistical difference between pretest and posttest tool application results, with postapplication testing showing higher effectiveness. This demonstrates the proposed mobile learning environment's efficiency, supporting motivation to learn using mobile technology. Recent studies in [54] confirm the mobile learning environment's effectiveness in providing blind students with specific skills and values. The improved psychosocial security marks among the pilot group students can be attributed to their exposure to the mobile learning environment. This improvement is noticed in their composition and future outlook, public and practical life, mood, social interactions, and relationships. Further gives credence to these results, showing a clear correlation between blind students' use of technology and their enhanced sense of psychological security, as depicted in Figure 3.

Figure 3 illustrates the noticeably elevated levels of psychosocial security in the experimental group's posttest measurement compared to their pretest scores.

For validating the results of the third imposition, stating “no statistically significant differences exist between the average scores of the experimental group in the posttest and follow-up measurements on the psychosocial security scale (dimensions and overall grade),” the Wilcoxon “*W*” test was applied. The results of this imposition can be seen in Table 13.

It is clear from the previous table that there are no statistically significant differences between the average grades of the experimental group members in dimensional and tracking measurements on the psychosecurity scale of blind students, i.e., there is a convergence between the average grades of the experimental group members in dimensional and tracking measurements on the psychosecurity scale of blind students and this validates the sixth imposition.

**Figure 4:**
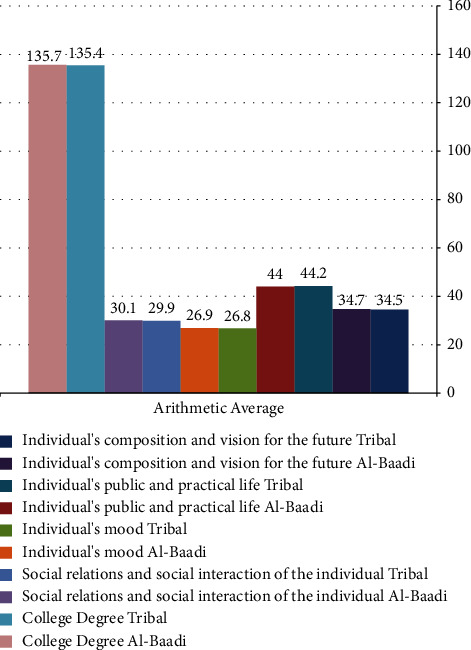
Intermediate dimensional and tracking measurements in the experimental group on the psychosecurity scale.

The study found that a mobile learning environment created through the Edmodo platform had a lasting and significant effect on improving the psychosocial security of blind university students. This was not a temporary improvement; the environment was systematically designed to ensure long-term benefits in enhancing psychosocial security, a crucial aspect of personality for blind individuals. Key features of this mobile learning environment included a variety of engaging digital content tailored to the needs of blind students, with a focus on auditory elements to accommodate their visual impairment. The environment supported unique educational elements such as accessibility, electronic access, repetition, and interactive discussions, along with feedback mechanisms to monitor students' progress in psychosocial security. The enduring positive impact of this mobile learning environment on the psychosocial security of the blind students in the study was evident and documented in the research.  Figure 4 illustrates the intermediate dimensional and tracking measurements obtained in the experimental group using the psychosecurity scale.

It is clear from the previous figure that there are no statistically significant differences in the psychosocial scores of students blind to the experimental group, indicating that the impact of the mobile learning environment remains with the experimental group, and given the previous findings, it is clear that the results of the current research can be disseminated to improve the psychosocial security of students blind to university education.

## 5. Limitations

This research, while pioneering in its efforts to enhance the digital and psychosocial security of blind university students using the Edmodo educational platform, encounters several limitations. The study's methodology, which combined descriptive and quasiexperimental approaches, was applied to a small sample size of 49 students from Beni Suef University. This small cohort may not adequately represent the broader population of blind students in higher education, thus limiting the generalizability of the findings. Additionally, the diversity of the student's academic disciplines introduces a level of variability that could influence the results. The research tools, including the Psychosecurity Measure, were originally designed for a broad spectrum of clinical and nonclinical groups and might not be entirely suitable for addressing the specific challenges and needs of the blind student demographic. The study's reliance on the Edmodo platform presupposes that all students have equal access to and are equally adept at using such technologies, which may not be the case in reality. Moreover, potential biases inherent in the research design, data collection, or analysis could have affected the outcomes. These factors underscore the necessity for further, more extensive research that would involve larger and more diverse samples to confirm the findings and recommendations of this study. Future research should also aim to address these potential biases and explore the effectiveness of mobile learning environments in reducing future anxiety and aiding in the vocational rehabilitation of blind students, ensuring that the benefits of such educational interventions can be extended to a wider audience with greater confidence.

## 6. Conclusions and Future Work

This research utilized a descriptive curriculum to determine the psychosocial security to be enhanced among blind university students. A quasiexperimental curriculum was employed to verify the impact of the independent variable “Mobile learning environment using Edmodo educational platform in affiliate variant” psychosocial security. The research was conducted on a sample of 49 blind students at Beni Suef University, with a focus on students from various colleges including Arts, Science with Special Needs, Media, Alson, and Law. The study also highlighted the demographics of these students and the various degrees they were pursuing. The research tools used in the study included a Psychosecurity Measure (Emotional Reassurance) Preparation, which was designed to diagnose psychological security for many diverse clinical groups in both health and disease, as well as in psychological and educational research. The Edmodo platform was also utilized as a free social education platform for teachers and students, with the potential for use by blind students. The goal of the research was to improve the digital and psychosocial security of blind university students, using the Edmodo platform to design a mobile learning environment. The results of the study were analyzed, and the psychosocial security of students was evaluated based on various aspects including their composition and vision for the future, their public and practical life, their mood, and their social relations and interactions. This research represents a significant step towards improving the digital and psychosocial security of blind students in higher education settings. The use of platforms like Edmodo not only facilitates learning but also provides a supportive environment for these students to interact and engage with their peers and educators. The findings of this study can be used as a basis for future research in this field, with a focus on further enhancing the digital and psychosocial security of blind students in higher education.

The researcher suggests some research that needs further study:

1. Mobile learning environments and their impact on improving the psychosocial security of blind students
2. The impact of mobile learning environments on reducing future anxiety in blind students
3. The role of mobile learning environments in the vocational rehabilitation of blind students

## Figures and Tables

**Table 1:** Recent studies on mobile learning environments for individuals with visual disabilities.

Author	Methodology	Results	Comment
Tan et al. [44]	A scoping review of 16,899 records from seven databases (CINAHL, Cochrane library, EMBASE, IEEE Xplore, Scopus, PubMed, Web of Science); 65 articles were included. Inclusion criteria: peer-reviewed articles in English from 2007 to 2021 discussing smartphone use by PVI, technologies for PVI, or training/support for PVI	48% focused on developing interfaces and apps for PVI. Only 5% discussed training/support for PVI. Smartphones and apps are effective and affordable AT for PVI. Many recent developments in smartphone technologies can further support PVI use. Specific smartphone features (zoom, magnification, and speech commands) are useful for different vision impairments	Highlights a significant gap in training/support for PVI, despite the importance of enhancing participation and independence. Recommends future research focus on evidence-based tailored training and support, especially in low- and middle-income countries. Suggests an individualized and graded approach to training based on the person's level of vision impairment and age. Emphasizes the importance of recognizing the steep learning curve for PVI, especially when transitioning to touchscreen phones
Akhmad et al. [45]	A descriptive qualitative approach with triangulation techniques in data collection. Data were collected from four blind students and four blind teachers at SLB 1 Makassar, using the context, input, process, product (CIPP) framework by Stufflebeam	Evaluates the unique learning dynamics at SLB 1 Makassar where both teachers and students are blind. Applications like be my eyes, voice dream reader, and blind Square significantly contribute to inclusive education and enhance the independence of blind children in science learning	Highlights the empathetic and understanding learning environment due to blind teachers teaching blind students. Notes the gap in technology availability and accessibility in schools and stresses the importance of these applications in fostering independence and effective learning
Kuriakose et al. [46]	Development and pilot testing of DeepNAVI, a smartphone-based navigation assistant leveraging deep learning. The system provides detailed information about obstacles, including type, position, distance, motion status, and scene information via audio mode	DeepNAVI offers comprehensive obstacle detection and information, ensuring portability and convenience without extensive training. Pilot tests with a user indicate the potential practicality and usefulness of the system for visually impaired individuals	Addresses gaps in existing navigation assistants by emphasizing portability, convenience, and detailed obstacle information. Highlights the system's small model size, rapid inference time, and effectiveness in real-time navigation for visually impaired users
Chen et al. [47]	Development and validation of the Apollo infant sight (AIS) system, a mHealth application that uses smartphone-based deep learning to identify visual impairments in children by analyzing gazing behaviors and facial features under visual stimuli. Data were collected from 3,652 children (≤48 months old)	AIS achieved an AUC of 0.940 in an internal validation set and 0.843 in an external validation set across multiple clinics in China. For at-home use by untrained parents or caregivers, AIS achieved an AUC of 0.859	The AIS system shows high potential for early detection of visual impairment in young children across a range of ophthalmic disorders. It can be effectively used by healthcare professionals and adapted for use by parents and caregivers at home

**Table 2:** The demographic characteristics of the research community.

Variables		College										Total
		Ethics		Special needs science		Media		Alsun		Law		
		Male	Female	Male	Female	Male	Female	Male	Female	Male	Female	
Bachelor's degree		13	13	—	—	1	2	2	6	1	1	39

Postgraduate	Public diploma	—	—	—	1	—	—	—	—	—	—	1
	Private diploma	—	—	—	1	—	—	—	—	—	—	1
	Master's degree	1	2	1	—	—	—	—	—	—	—	4
	Ph.D.	—	2	1	—	—	1	—	—	—	—	4

Total		14	17	2	2	1	3	2	6	1	1	49

**Table 3:** Average age of the search sample.

The average age of the search sample			
Category	Number	Middle age	Standard deviation
Male	9	19.11	2.32
Female	11	19.83	3.56
Total	20	19.52	3.015

**Table 4:** Quasiexperimental design.

Groups	Tribal application	Quasi-experimental processing	Remote application	Tracking application
Experimental	Psychosecurity measure	X	Psychosecurity measure	Psychosecurity measure
Officer		Y		

**Table 5:** Parity between groups (experimental and control) of time age.

Group	*N*	Arithmetic average	Standard deviation	Average grades	Total grades	*u*	*z*	Indicative level
Experimental	10	19.7	3.68	10.3	103	48	0.153	0.912 Irrelevant
Officer	10	19.35	2.35	10.7	107			

**Table 6:** Parity between the two groups (experimental and control) in psychosocial security.

Dimensions									
Psychosocial security associated with	Group	*N*	Arithmetic average	Standard deviation	Average grades	Total grades	*U*	*z*	Indicative level
Individual's composition and vision for the future	Experimentation	10	22.6	6.1	10.25	102.5	47.5	0.19	0.853
Irrelevant									
Officer	10	22.9	6.35	10.75	107.5				

Individual's public and practical life	Experimental	10	31.2	8.52	10.3	103	48	0.152	0.912
Irrelevant									
Officer	10	31.5	5.36	10.7	107				

Individual's mood	Experimentation	10	19	5.66	11.3	113	42	0.606	0.579
Irrelevant									
Officer	10	17.8	6.05	9.7	97				

Social relations and social interaction of the individual	Experimentation	10	17.7	5.66	9.55	95.5	40.5	0.72	0.481
Irrelevant									
Officer	10	19.8	4.61	11.45	114.5				

College degree	Experimentation	10	90.5	17	9.55	95.5	40.5	0.719	0.481
Irrelevant									
Officer	10	92	13.4	11.45	114.5				

**Table 7:** Correlation factors between individual grades and the overall degree of their dimension.

m.	Binding coefficient	m.	Binding coefficient	m.	Binding coefficient	m.	Binding coefficient
Individual's composition and vision for the future	Individual's public and practical life	15	0.444^*∗*^	Relationships and social interaction of the individual			
1	0.521^*∗∗*^	1	0.883^*∗∗*^	16	0.446^*∗*^	1	0.539^*∗∗*^
2	0.630^*∗∗*^	2	0.531^*∗∗*^	17	0.550^*∗∗*^	2	0.439^*∗*^
3	0.422^*∗*^	3	0.624^*∗∗*^	18	0.431^*∗*^	3	0.561^*∗∗*^
4	0.655^*∗∗*^	4	0.408^*∗*^	Individual's mood		4	0.612^*∗∗*^
5	0.482^*∗*^	5	0.542^*∗∗*^	1	0.693^*∗∗*^	5	0.307^*∗∗*^
6	0.583^*∗∗*^	6	0.722^*∗∗*^	2	0.699^*∗∗*^	6	0.548^*∗∗*^
7	0.442^*∗*^	7	0.547^*∗∗*^	3	0.407^*∗*^	7	0.506^*∗∗*^
8	0.646^*∗∗*^	8	0.903^*∗∗*^	4	0.653^*∗∗*^	8	0.530^*∗∗*^
9	0.794^*∗∗*^	9	0.720^*∗∗*^	5	0.443^*∗*^	9	0.509^*∗∗*^
10	0.635^*∗∗*^	10	0.452^*∗*^	6	0.399^*∗*^	10	0.518^*∗∗*^
11	0.583^*∗∗*^	11	0.590^*∗∗*^	7	0.569^*∗∗*^	11	0.543^*∗∗*^
12	0.892^*∗∗*^	12	0.455^*∗*^	8	0.633^*∗∗*^	12	0.430^*∗*^
13	0.629^*∗∗*^	13	0.662^*∗∗*^	9	0.643^*∗∗*^		
14	0.619^*∗∗*^	14	0.610^*∗∗*^	10	0.618^*∗∗*^		

^*∗*^Function at an indicative level of 0.01; ^*∗∗*^Function at an indicative level of 0.05.

**Table 8:** Psychosecurity scale associations' matrix.

m.	Dimensions	1	2	3	4	College
1	Individual's composition and vision for the future	—				
2	Individual's public and practical life	0.703^*∗∗*^	—			
3	Individual's mood	0.501^*∗∗*^	0.563^*∗∗*^	—		
4	Social relations and social interaction of the individual	0.553^*∗∗*^	0.552^*∗∗*^	0.535^*∗∗*^	—	
College degree	0.669^*∗∗*^	0.749^*∗∗*^	0.554^*∗∗*^	0.584^*∗∗*^	—	

^*∗∗*^D at an indicative level (0.01).

**Table 9:** Psychosecurity scale stabilizers using the application method and reapplication.

Scale dimensions	The correlation coefficient between the first and second applications	Indicative level
Individual's composition and vision for the future	0.793	0.01
Individual's public and practical life	0.611	0.01
Individual's mood	0.751	0.01
Social relations and social interaction of the individual	0.591	0.01
College degree	0.531	0.01

**Table 10:** Psychosecurity scale stabilizers using the Alpha-Cronbach coefficient.

m	Scale dimensions	Alpha-Cronbach coefficient
1	Individual's composition and vision for the future	0.718
2	Individual's public and practical life	0.672
3	Individual's mood	0.711
4	Social relations and social interaction of the individual	0.678
College degree		0.791

**Table 11:** Mann–Whitney test results and *Z* value indicating differences in average scores of dimensional measurement between experimental and control groups on the psychosecurity scale.

Dimensions	Group	*N*	Arithmetic average	Standard deviation	Average grades	Total grades	*z*	Indicative level
Individual's composition and vision for the future	Experimental	10	34.5	5.08	14.7	147	3.181	0.01
	Officer	10	23.7	5.91	6.3	63		

Individual's public and practical life	Experimental	10	44.2	6.21	15.4	154	3.712	0.01
	Officer	10	28.5	5.62	5.6	56		

Individual's mood	Experimental	10	26.8	2.35	14.65	146.5	3.157	0.01
	Officer	10	18.4	5.42	6.35	63.5		

Social relations and social interaction of the individual	Experimental	10	29.9	3	15.2	152	3.57	0.01
	Officer	10	19	5.48	5.8	58		

College degree	Experimental	10	135.4	12.02	15.5	155	3.781	0.01
	Officer	10	89.6	13.57	5.5	55		

**Table 12:** Wilcoxon test results and *Z* value indicating differences in average scores of tribal and postmeasurements in the experimental group on the psychosecurity scale.

Dimensions	*N*	Measurement	Arithmetic average	Standard deviation	Tribal/remote measurement	Number	Average ranks	Total grades	*z* value	Connectedness
Individual's composition and vision for the future	10	Tribal	22.60	6.10	Negative grades Positive grades Equality Total	1 9 0 10	1.00 6.00	1.00 54.00	2.703	0.01
	10	Al-Baadi	34.50	5.08						

Individual's public and practical life	10	Tribal	31.20	8.52	Negative grades Positive grades Equality Total	2 8 0 10	1.50 6.50	3.00 52.00	2.501	0.01
	10	Al-Baadi	44.20	6.21						

Individual's mood	10	Tribal	19.00	5.66	Negative grades Positive grades Equality Total	1 9 0 10	3.00 5.78	3.00 52.00	2.502	0.01
	10	Al-Baadi	26.80	2.35						

Social relations and social interaction of the individual	10	Tribal	17.70	5.66	Negative grades Positive grades Equality Total	0 10 0 10	0.00 5.50	0.00 55.00	2.805	0.01
	10	Al-Baadi	29.90	3.00						

College degree	10	Tribal	90.50	17.00	Negative grades Positive grades Equality Total	1 9 0 10	1.00 6.00	1.00 54.00	2.703	0.01
	10	Al-Baadi	135.40	12.02						

**Table 13:** Wilcoxon test results and *Z* value indicating differences in average scores of dimensional and tracking measurements in the experimental group on the psychosecurity scale.

Dimensions	*N*	Measurement	Arithmetic average	Standard deviation	Tribal/remote measurement	Number	Average ranks	Total grades	*z* value	Connectedness
Individual's composition and vision for the future	10	Tribal	34.50	5.08	Negative grades Positive grades Equality Total	5 5 0 10	4.50 6.50	22.50 32.50	0.540	0.589 Irrelevant
	10	Al-Baadi	34.70	4.32						

Individual's public and practical life	10	Tribal	44.20	6.21	Negative grades Positive grades Equality Total	5 5 0 10	6.00 5.00	30.00 25.00	0.277	0.782 Irrelevant
	10	Al-Baadi	44.00	5.98						

Individual's mood	10	Tribal	26.80	2.35	Negative grades Positive grades Equality Total	5 5 0 10	5.00 6.00	25.00 30.00	0.277	0.782 Irrelevant
	10	Al-Baadi	26.90	2.42						

Social relations and social interaction of the individual	10	Tribal	29.90	3.00	Negative grades Positive grades Equality Total	5 5 0 10	4.50 6.50	22.50 32.50	0.540	0.589 Irrelevant
	10	Al-Baadi	30.10	3.31						

College degree	10	Tribal	135.40	12.02	Negative grades Positive grades Equality Total	5 5 0 10	4.60 6.40	23.00 32.00	0.471	0.638 relevant
	10	Al-Baadi	135.70	10.70						

## Data Availability

The data that support the findings of this study are available from the corresponding author upon reasonable request.
